# Modulatory Effects of Chinese Herbal Medicines on Energy Metabolism in Ischemic Heart Diseases

**DOI:** 10.3389/fphar.2020.00995

**Published:** 2020-07-03

**Authors:** Fanghe Li, Jinmao Li, Saisai Li, Shuwen Guo, Ping Li

**Affiliations:** ^1^ The 3rd Affiliated Hospital, Beijing University of Chinese Medicine, Beijing, China; ^2^ Fangshan Hospital, Beijing University of Chinese Medicine, Beijing, China

**Keywords:** energy metabolism, ischemic heart disease, Chinese herbal medicines, cardiac metabolism, mitochondrial function

## Abstract

Ischemic heart disease (IHD), a major global public health problem, is associated with high morbidity and mortality. Although the very best of modern approaches have proven effective in reducing morbidity and mortality, the poor prognosis of patients with IHD remains a major clinical concern. Cardiac energy metabolism is increasingly recognized as having a role in the pathogenesis of IHD, inducing metabolic substrate alterations, mitochondrial dysfunction, impaired function of the mitochondrial electron transport chain, and deprivation of cardiac energy. Factors involved in cardiac energy metabolism provide potential therapeutic targets for the treatment of IHD. Chinese herbal medicines (CHMs) have a long history of use in the prevention and treatment of cardiovascular diseases with multi-component, multi-target, and multi-signaling. Increasing evidence suggests that Chinese herbal medicines may improve myocardial ischemia through modulating cardiac energy metabolism. Here, we describe the possible targets and pathways of cardiac energy metabolism for CHMs, and appraise the modulatory effects of CHMs on energy metabolism in IHD. Especially, this review focuses on summarizing the metabolic effects and the underlying mechanisms of Chinese herbal medicines (including herbs, major bioactive components, and formulas) in IHD. In addition, we also discuss the current limitations and the major challenges for research investigating the use of CHMs in the treatment of cardiovascular diseases.

## Introduction

Ischemic heart disease (IHD) is the most common cause of death among cardiovascular diseases, which imposes a substantial social and economic burden. The Global Burden of Disease Study of 2017 (GBD 2017) has reported that the total number of deaths from IHD increased from 7.30 to 8.93 million between 2007 and 2017 at a global level (GBD 2017 Causes of Death Collaborators, 2018). IHD is comprised principally of coronary heart disease (including angina, nonfatal myocardial infarction, and coronary death), asymptomatic myocardial ischemia, sudden cardiac death, and ischemic heart failure (Wong, 2014; Guo et al., 2018). Current therapeutic approaches are mainly dependent on medical interventions such as statins, antiplatelet drugs, beta-receptor blockers (β-blockers), and angiotensin-converting-enzyme inhibitors (ACEIs), in addition to surgical procedures such as percutaneous coronary intervention (PCI) and coronary artery bypass graft (CABG) surgery. Although these medical and surgical therapies have proven effective in reducing morbidity and mortality after IHD, millions of patients still have clinical symptoms, including chest tightness, heart palpitations, shortness of breath, and fatigue. Therefore, it is crucial to develop novel treatment strategies involving different mechanisms in myocardial ischemia and even reperfusion.

Cardiac energy metabolism plays a major role in the progression of cardiovascular diseases. Van Bilsen et al. (2004) proposed the concept of myocardial metabolic remodeling. With the development of modern science and advanced technologies, alterations in myocardial energetics such as shifts in energy substrate utilization, impaired mitochondrial oxidative phosphorylation, and reduction in the adenosine triphosphate (ATP) transfer and utilization capacity are increasingly recognized as playing a crucial role in the mechanisms of IHD (Fukushima et al., 2015; Tuomainen and Tavi, 2017). Deprivation of cardiac energy leads to cardiac contractile dysfunction, left ventricular remodeling, and even heart failure (HF). Consequently, growing evidence supports that modulation of cardiac energy metabolism can be an effective means of improving cardiac function and slowing the progression to HF (Neubauer, 2007; Lang et al., 2015; Qi and Young, 2015; Yang et al., 2016; Tuomainen and Tavi, 2017). Chinese herbal medicines (CHMs) have drawn much attention recently as a potential therapeutic strategy for the prevention and treatment of myocardial ischemia through modulating energy metabolism. It is a novel strategy for protecting the ischemic myocardium against IHD. This review focuses on the potential efficacy of herbs, major bioactive component (MBC), and Chinese herbal formulas (CHF) in modulating cardiac energy metabolism in IHD, as well as the associated mechanisms.

## Targets and Signaling of Cardiac Energy Metabolism for Chinese Herbal Medicines

### “Qi-blood” Theory of TCM Is Connected With Cardiac Energy Metabolism

The healthy adult heart has perpetually high energy demands, and needs to continuously contract to supply the body with blood and oxygen. As powerhouses of cardiomyocytes, mitochondria are continuously supplying the energy required for cardiac muscle contraction. Under normal conditions, almost of ATP generation in the healthy adult heart comes from mitochondrial oxidative phosphorylation, with the rest mainly derived from glycolysis. In ischemic heart, impaired mitochondrial oxidative phosphorylation provides an insufficient supply of ATP to cardiomyocytes. The available evidence suggests that cardiac energy metabolism is in good correlation with cardiac function. Reduced capacity for cardiac energy transduction leads to cardiac pump dysfunction, blood flow disturbance, cardiac contractile dysfunction, and even heart failure (Huss and Kelly, 2005). The search for treatment strategies for modulating cardiac energy metabolism is one of the major challenges in cardiovascular diseases.

Traditional Chinese medicine (TCM) is characterized by a “Holistic concept” that the organism is considered as a whole. In TCM, Qi and blood are the essential substances of organisms, which maintain the life activity of human. Qi has promoting, warming, consolidation, and retention function, which provides energy for promoting blood circulation and keeps incessantly the blood flowing within the vessels. As the first Chinese medical classic and the origin of TCM theory, the *Suwen* of *Yellow Emperor’s Internal Classic* describes that heart governing blood and vessels. It means that Heart-Qi promotes and keeps the formation and circulation of blood in the vessels for nourishing the organs and tissues, retaining body fluid balance, and maintaining the normal physiological activities. An abundance of the Heart-Qi, a sufficiency of blood, and vascular patency are three principal components that control the normal circulation of blood. In the heart, Heart-Qi drives ATP synthesis *via* ATP synthase in the cardiac mitochondria to provide the vital energy necessary for cardiac muscle contraction and relaxation. Symptoms of myocardial ischemia in clinical patients mainly include chest tightness, heart palpitations, shortness of breath, and weakness. These symptoms of myocardial ischemia correspond to the symptoms of Heart Qi deficiency syndrome, which further cause blood circulation disorder and cardiac microcirculatory disturbance leading to blood stasis syndrome. Deficiency of Heart Qi can also cause insufficiency of the Heart-Yang, which is accompanied by a series of symptoms such as cold sweat, intolerance to cold and cold limbs. Moreover, Heart Qi deficiency can induce microvascular hyperpermeability, leading to excessive fluid, phlegm, edema, and hemorrhage. Based on the “Qi-blood” theory of TCM, Chinese herbal medicines that can tonify or regulate Qi and activate blood have promise as an important therapeutic approach to the modulation of cardiac energy metabolism in cardiology.

### The Possible Targets of Cardiac Energy Metabolism for Chinese Herbal Medicines

**Figure 1:**
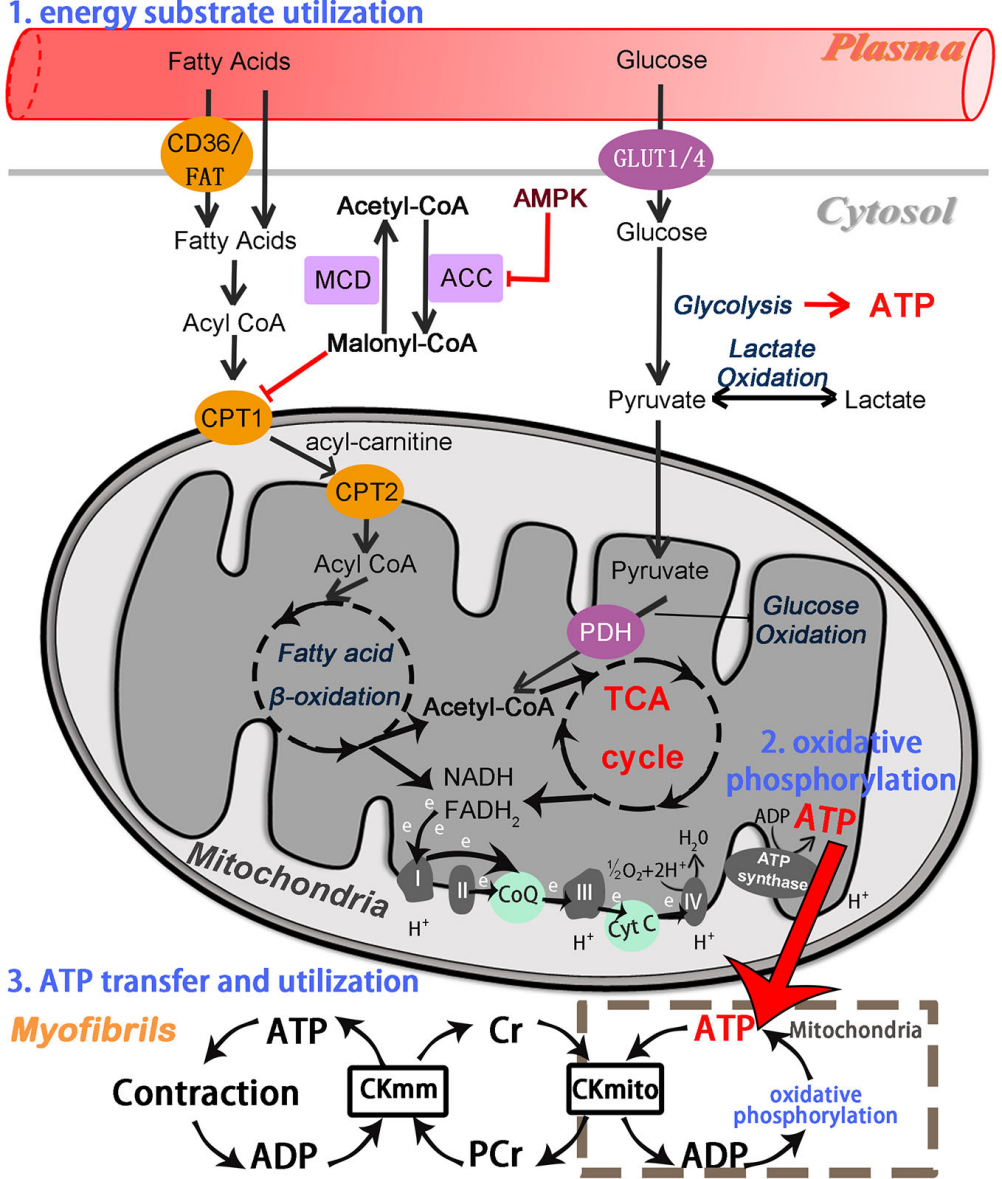
Overview of the cardiac energy metabolism. Fatty acid beta-oxidation and glucose oxidation in mitochondria, respectively, yield acetyl-CoA, which is fed into the TCA cycle to produce NADH and PADH_2_. These further enter the mitochondrial electron transport chain and then drive ATP synthesis. GLUT, glucose transporter; CPT1 and CPT2, carnitine palmitoyltransferase 1 and 2; PDH, pyruvate dehydrogenase; TCA, tricarboxylic acid cycle; NADH, nicotinamide adenine dinucleotide; FADH_2_, flavin adenine dinucleotide; CoQ, coenzyme Q (ubiquinone); Cyt C, cytochrome C; ATP, adenosine triphosphate; ADP, adenosine diphosphate; H^+^, hydrogen ion; PCr, phosphocreatine; Cr, creatine; CKmito, mitochondrial creatine kinase; CKmm, myofibrillar creatine kinase.

Chinese herbal medicines, as natural botanical herbs, have a long history of clinic use in the treatment of cardiovascular diseases, and have properties of numerous potential pharmacological targets. Them hold the great and unique potential in the management of cardiac energy metabolism, especially in the aspects of mitochondrial function, lipid metabolism, and glucose metabolism. Some of these possible targets are described below, categorized by the process of cardiac energy metabolism. The metabolic process involved in cardiac energy metabolism consists of three main components (**Figure 1**), namely, energy substrate preference, mitochondrial oxidative phosphorylation, and ATP transfer and utilization (Neubauer, 2007).

Energy substrate utilization represents the first component. Cardiomyocytes can metabolize all classes of energy substrates, including fatty acids, glucose, glycogen, lactate, ketone bodies, and certain amino acids (Heggermont et al., 2016). Free fatty acids (FFA) and glucose first enter the myocardium from the plasma, and are then converted respectively to fatty acyl-coenzyme A (acyl-CoA) and glycolytic end product pyruvate in the cytoplasm of cardiomyocytes. Long-chain fatty acyl-CoA is transported into mitochondria *via* carnitine palmitoyl transferase 1 and 2 (CPT1 and CPT2), whereas pyruvate is taken up into mitochondria by the mitochondrial pyruvate carrier (MPC) (Arumugam et al., 2016; Noordali et al., 2018).

The second component is mitochondrial oxidative phosphorylation, which supplies more than 95% of the ATP required by the mature heart. Normally, fatty acid beta-oxidation (FAO), the major source of mitochondrial oxidative phosphorylation, provides more than two-thirds of the energy demands in adult myocardium, with the remainder being provided by the oxidation of substrates such as carbohydrates, lactate, ketone bodies, and several amino acids (Heggermont et al., 2016). These mitochondrial substrate fluxes *via* specific metabolic steps (especially fatty acid beta-oxidation and pyruvate oxidation) yield acetyl coenzyme A (acetyl-CoA), which subsequently enters the tricarboxylic acid (TCA) cycle (Kolwicz et al., 2013). Nicotinamide adenine dinucleotide (NADH) and flavin adenine dinucleotide (FADH_2_) are generated by the TCA cycle and beta-oxidation, respectively (Schwarz et al., 2014). NADH and FADH_2_ feed high energy electrons into the mitochondrial electron transport chain (ETC), generating an electrochemical gradient through ETC complexes (complex I-V) across the inner mitochondrial membrane (IMM) that subsequently drives ATP synthesis (Huss and Kelly, 2005). Among them, ATP synthase (complex V), as the final step of mitochondrial oxidative phosphorylation, generates ATP by phosphorylating adenosine diphosphate (ADP). The transfer of electrons between complexes is mediated by ubiquinone (CoQ) and cytochrome c (cyt c). As well as generating NADH and FADH_2_, the TCA cycle also produces excess citrate in the cytosol, where it is converted into acetyl CoA (Murphy et al., 2016; Noordali et al., 2018). Cytosolic acetyl CoA is further converted into malonyl CoA *via* acetyl CoA carboxylase (ACC), whereas malonyl CoA, a potent inhibitor CPT-1, can be converted back into acetyl CoA *via* malonyl CoA decarboxylase (MCD), thereby regulating the entry of FFA into the mitochondria once again (Fukushima et al., 2015; Noordali et al., 2018). The third component comprises cardiac ATP transfer and utilization *via* the creatine kinase (CK) system (Neubauer, 2007; Fukushima et al., 2015). High-energy phosphates are transferred from the ATP generated *via* oxidative phosphorylation in the mitochondria to creatine (Cr), thus forming phosphocreatine (PCr) and ADP by the action of mitochondrial creatine kinase. Phosphocreatine rapidly diffuses from the mitochondria into the myofibrils and then reforms ATP and Cr *via* the action of myofibrillar creatine kinase (Neubauer, 2007). Subsequently, ATP is used by myosin ATPase to produce the force of cardiac contraction, while the free Cr diffuses back to the mitochondria.

### The Possible Transcriptional Signaling of Cardiac Energy Metabolism for Chinese Herbal Medicines

The mechanisms of cardiac energy metabolism are complex, and are primarily controlled by metabolic proteins (enzymes and transcriptional components) that regulate the expression of a large number of genes involved in myocardial energy metabolism through multiple metabolic pathways (Stanley et al., 2005). In particular, mitochondrial structure and function are regulated by numerous genes, including the 37 encoded in mitochondrial DNA and a considerable number encoded in nuclear DNA (Ham and Raju, 2016). It is becoming increasingly clear that multiple nuclear-mitochondrial crosstalk and signaling pathways play an important role in regulating cardiac energy metabolism under ischemic conditions (Qi and Young, 2015; Murphy et al., 2016).

**Figure 2:**
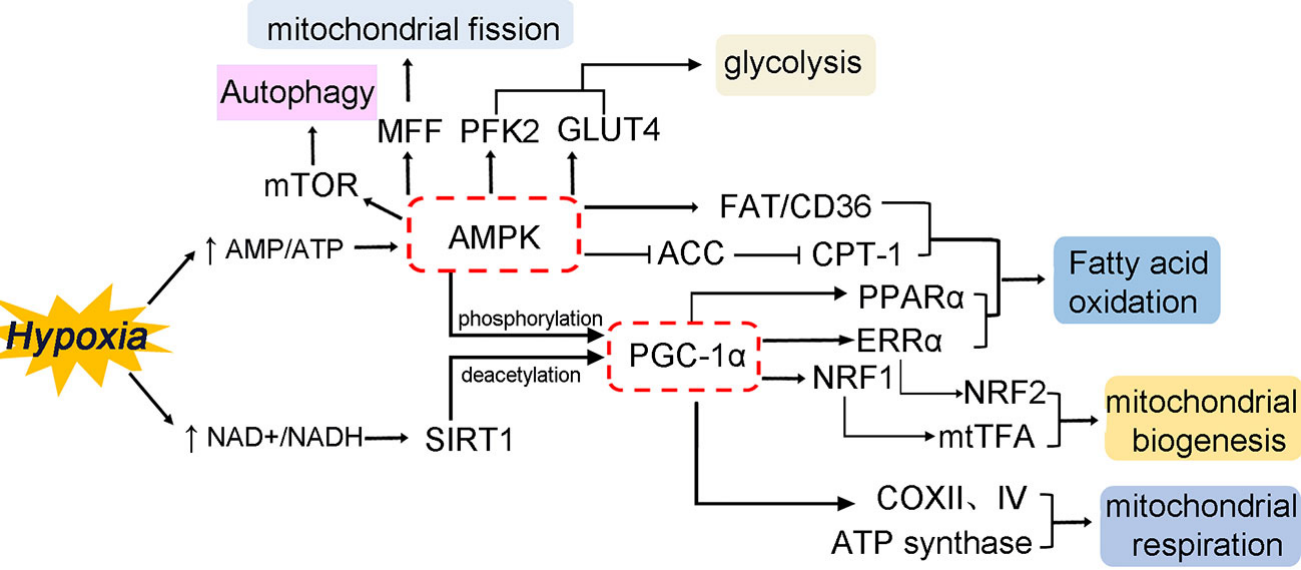
Transcriptional control of cardiac energy metabolism in hypoxia/ischemia. The figure mainly summarizes the modulatory effects of AMPK and PGC-1α by multiple metabolic pathways and its interactions with nuclear receptors and transcription factors to regulate cardiomyocyte metabolism. The multiple physiological processes involve multiple aspects, including substrate preference, mitochondrial biogenesis, mitochondrial dynamics, mitochondrial autophagy, and mitochondrial respiration.

Chinese Herbal Medicines can also modulate numerous potential pathways because of their properties of multi-component. Some of these possible pathways are described below (**Figure 2**). Adenosine monophosphate-activated protein kinase (AMPK) is a critical intracellular energy sensor, and its activation is involved in multiple signaling pathways, including modulating glucose and fatty acid metabolism, mitochondrial function, and autophagy (Murphy et al., 2016; Nishida and Otsu, 2016). AMPK consists of three protein subunits: a catalytic α subunit, containing the Thr^172^ site that must be phosphorylated for AMPK activation, and two regulatory subunits (γ and β) (Zaha and Young, 2012). The AMPK activity is partly activated by an increase in AMP/ATP ratio in low-energy states. During myocardial ischemia, the activity of AMPK in myocardium is activated as an adaptive response to cardiomyocyte stress, leading to a series of changes in metabolic pathways. Activation of AMPK increases cellular glucose uptake by mediating the transport of the glucose transporter 4 (GLUT4) from the cytosol to the sarcolemma membrane in ischemia at an early adaptive stage (Russell et al., 2004; Qi and Young, 2015), and promotes glycolysis through phosphofructokinase 2 (PFK2) phosphorylation (Marsin et al., 2000). AMPK can inhibit the activity of glycogen synthase (GS), which indirectly promotes glycogen utilization (Qi and Young, 2015). Moreover, AMPK also plays a critical role in modulating lipid metabolism. Activated AMPK facilitates the myocardial uptake of fatty acids by promoting the translocation of the fatty acid transporter CD36 (Luiken et al., 2003). Meanwhile, Activation of AMPK further results in decrease of malonyl-CoA levels *via* inactivation of ACC, which effectively promotes fatty acid oxidation by relieving CPT-1 suppression (Dyck and Lopaschuk, 2006) (**Figure 1**). Meanwhile, the process of mitochondrial biogenesis keeps in a dynamic balance, which undergoes through constant fusion and fission. Dynamin-related protein 1 (Drp1) and Fission 1 (Fis1) are known to promote mitochondrial fission. Mitofusin 1 and 2 (MFN1 and MFN2) mainly mediate outer membrane fusion, whereas Opa1 is mainly responsible for inner membrane fusion. Mitochondrial dynamics imbalance leads to defects in mitochondrial morphology and mitochondrial dysfunction during ischemic contexts. Hypoxia-induced AMPK activation can promote mitochondrial fission *via* the phosphorylation of mitochondrial fission factor (MFF), which is considered to be a mitochondrial outer membrane receptor for Drp1, an essential enzyme for providing a driving force in mitochondrial fission (Garcia and Shaw, 2017). Besides, autophagy is regulated by AMPK activation, which restores impaired myocardial function *via* mechanistic target of rapamycin (mTOR) (Wu et al., 2020a).

Peroxisome proliferator-activated receptor gamma (PPARγ) coactivator (PGC-1α) is a well-characterized mediator of mitochondrial biogenesis and respiratory, and its activity can also be modulated by AMPK phosphorylation (Gundewar et al., 2009) (**Figure 2**). In addition to AMPK phosphorylation, PGC-1α activity is tightly controlled by the NAD^+^-dependent deacetylase sirtuin-1 (SIRT1) deacetylation, which promotes mitochondrial biogenesis (Fernandez-Marcos and Auwerx, 2011; Zaha and Young, 2012; Ham and Raju, 2016). As a cofactor, PGC-1α is known to control the expression of multiple nuclear receptors and transcription factors, thereby regulating the entire metabolic phenotype of cardiomyocytes. PGC-1α modulates mitochondrial biogenesis and oxidative phosphorylation by directly activating nuclear respiratory factors (NRF1 and NRF2) and the estrogen-related receptor alpha (ERRα) transcription factor. NRF1 activates the downstream synthesis of mitochondrial transcription factor A (mtTFA), which regulates mtDNA replication, transcription, and maintenance (Kang and Hamasaki, 2005; Rowe et al., 2010). As a major transcriptional partner of PGC-1α, ERRα can induce an increase in the expression of NRF2, modulating cardiomyocytes cycle and differentiation, and mitochondrial biogenesis (Ham and Raju, 2016). PGC-1α also co-activates PPARα, which involves in fatty acid metabolism in cardiomyocytes (Finck, 2007; Lehman et al., 2000). Furthermore, PGC-1α activation enhances mitochondrial respiration through increasing the expression of cytochrome c, cytochrome c oxidase subunits II and IV (COX II and IV), and ATP synthase (Choi et al., 2008; Espinoza et al., 2010).

## Modulatory Effects of Chinese Herbal Medicines on Energy Metabolism in IHD

Cardiac energy metabolism is highly flexible with respect to energy substrates, with a dynamic balance that is modified by aging, as well as physiological and pathological contexts (Huss and Kelly, 2005; Arumugam et al., 2016). Increased fatty acid beta-oxidation with aging is accompanied by a progressive decrease in glycolytic metabolism. The fetal heart uses glucose oxidation as a major source of energy, while the adult myocardium is considerably more dependent on fatty acid metabolism. Interestingly, during ischemic conditions, the cardiac metabolic profile shows significant similarities with that of the fetus. This phenomenon is considered to revert to the “fetal phase” (Tuomainen and Tavi, 2017). In addition to shifts in cardiac substrate utilization, alterations in mitochondrial ultrastructural and function play a crucial role in the mechanisms of IHD. Cardiac mitochondria, as the powerhouses of the cardiomyocytes, involve a complex series of processes of oxidative phosphorylation. Them are not only a primary source of ATP synthesis and reactive oxygen species (ROS) production in cardiac myocytes, but also play a critical role in the process of apoptosis. Myocardial hypoxia/ischemia inhibits a series of processes of mitochondrial oxidative phosphorylation and diverts the pyruvate to lactate leading to cellular acidification. The ischemic cardiomyocyte shows a marked reduced ability to synthesize ATP, a significant increased mitochondrial ROS production, calcium influx, and even Ca^2+^ overload leading to mitochondrial membrane permeability transition, loss of the mitochondrial membrane potential (MMP) and mitochondrial swelling with the release of cytochrome c. These phenomena further cause the apoptosome activation and caspase-mediated apoptosis (Ham and Raju, 2016). At reperfusion, there occurs a series of mitochondrial derangements, including the rapid re-establishment of oxidative phosphorylation, inhibition of respiratory chain activity, mitochondrial ROS accumulation, and Ca^2+^ overload, mitochondrial membrane permeability transition pore (mPTP) opening, mitochondrial-dependent apoptosis, and even cell death (Ham and Raju, 2016; Wu et al., 2020a).

Modern therapies, such as ACEIs and beta-blockers, have indirect effects on cardiac metabolism in addition to their classical effects, but them do not directly influence cardiac energy metabolism (Neubauer, 2007). Growing evidence suggests that the modulation of cardiac metabolism may be the promising therapeutic approach in patients with IHD (Noordali et al., 2018; Doehner et al., 2014; Heggermont et al., 2016). The known metabolic modulators such as Trimetazidine, L-Carnitine, and Coenzyme Q10 are currently used in clinical trials. The metabolic mechanisms of these modulators mainly involve inhibition of fatty acid oxidation, stimulation of glucose oxidation, and protection of mitochondrial function (Suner and Cetin, 2016; Di Napoli et al., 2007; Xue et al., 2007; Fotino et al., 2013). In TCM, Chinese herbal medicines are widely used in the treatment of cardiovascular diseases in clinic. CHMs have their own advantages that are due to pharmacological properties of multi-component, multi-target, and multi-pathway. An increasing number of studies have shown that CHMs with replenishing Qi or Yang and activating blood or resolving blood stasis can regulate cardiac energy metabolism in IHD (Wong and Ko, 2013; Chen et al., 2015; Zhang et al., 2013; Li et al., 2018a).

In this article, we mainly summarize the metabolic effects and underlying mechanisms of Chinese herbal medicines, major bioactive component of CHMs, and Chinese herbal formulas in IHD, respectively (**Tables 1** and **2**). In particular, the model of acute myocardial infarction is usually induced by left anterior descending (LAD) coronary artery ligation, which is most widely used surgical animal model. Isoproterenol (Iso)-induced myocardial infarction model is a well-developed non-surgical MI model (Kumar et al., 2016). Therefore, the major inclusion criteria included Iso-included MI model, LAD coronary artery ligation-induced MI model, and myocardial ischemia and reperfusion (I/R) injury model. The major exclusion criteria included exercise training, metabolomics analysis, angiotensin II-induced HF model, abdominal aorta ligation-induced HF model, cobalt chloride-induced myocardial ischemia, and doxorubicin-induced myocardial injury.

**Table 1:** Metabolic effect and mechanism of CHMs and the major bioactive component in IHD.

Source	Compound	Frequency (n)	Dosage	Effects/Mechanism of action	References
*Astragalus mongholicus Bunge*	Extract	1	100 mg/kg/day, 200 mg/kg/day	↓LDH, FFA, PA, and LA, ↑ATP, ADP, and TAN	Jin et al., 2014
	Astragalus polysaccharides	1	80 mg/kg/day, 100 μg/ml	↓ANP and FFA, ↑ATP/ADP and ATP/AMP, ↑ATP5D, PGC-1α, and PDK4, ↓TNF-α	Luan et al., 2015
	Astragalosides	1	5 mg/kg/day	↑ATP, PCr, and Cr, and regulating intracellular Ca^2+^ homeostasis	Chen et al., 2006
		3	10 mg/kg/day	↑cTnI, ↑ATP/ADP, and ATP/AMP, ↑ATP5D, and Complex V	Cui et al., 2018
			10 mg/kg/day	↑cTnI, ↑ATP/ADP, and ATP/AMP, ↑ATP5D, and p-MLC-2, ↑Complex V, ↓Bax/Bcl-2	Tu et al., 2013
			80 mg/kg/day	↑ATP/AMP, ↓ANP, BNP, and FFA, ↑ATP5D, PGC-1α, ↓p65	Zhang et al., 2015
*Astragalus mongholicus Bunge*; *Panax ginseng C.A.Mey.*	kaempferol	1	20 μM	↓LDH and ROS levels, ↓the loss of ΔΨm, and the release of cytochrome c from mitochondria into cytosol, ↓mPTP opening, ↑SIRT1	Guo et al., 2016
*Astragalus mongholicus Bunge*	Formononetin	1	5, 10, and 30 μM	↓ROS, ↓the loss of mitochondrial membrane integrity and mPTP opening, ↑Akt activation, and GSK-3β (Ser9) phosphorylation	Cheng et al., 2016
*Panax ginseng C.A.Mey.*; *Panax Notoginseng (Burkill) F.H.Chen*	Ginsenoside Rb1	3	5 mg/kg/h	↓cTnI, ↑ATP, ATP/ADP, and ATP/AMP, ↑ATP5D, and ATP synthase, ↓RhoA, ↓p-MYPT/MYPT, and p-MLC/t-MLC	Cui et al., 2017
			100 μmol/L	↓mPTP opening and restored loss of mitochondrial membrane potential, ↑p-AKT/AKT, p-GSK-3β/GSK-3β	Li et al., 2016b
			5 mg/kg/h	↑ATP/ADP, and ATP/AMP, ↑ATP5D, and ATP synthase	Cui et al., 2018
	Ginsenoside Rg1	2	5 mg/kg/h	↓cTnI, ↓ADP/ATP, and AMP/ATP, regulation of energy metabolism-related proteins such as ↓ALDOA, ENOα, and HIF1α, ↑ECH1, ENOβ, ATP5D, COXI, and COXII, ↑ATP synthase activity, ↓RhoA/ROCK activation, ↓p-RhoA, ↓p-MLC	Li et al., 2018b
			12.5 μM	↑OCRs, ↑mitochondrial length, and MMP, ↓ROS and GDH, ↑MFN2, Rg1 had no significant effect on MFN1, OPA1, and Drp1	Dong et al., 2016
*Panax ginseng C.A.Mey.*	Ginsenoside Rd	1	10 μM	↓LDH and CK, ↑MMP, ↓ROS, ↓cytosolic translocation of mitochondrial cytochrome c, ↑AKT, and GSK-3β phosphorylation	
	Ginsenoside Rg5	1	50 mg/kg/d; 10 μM	↑ATP and MMP, ↓lactate content and NADH/NAD^+^ratio, CPT-1A, p-PDH, and OCRs, ↑p-AKT (Ser473), ↑HK-II binding to mitochondria and ↓HK-II expression in the cytosol,↓Drp1 recruitment to mitochondria	Yang et al., 2017c
	Panax ginseng Polysaccharide	1	200 mg/ml	↑ATP, ↑MMP, ↓the release of cytochrome c, ↑oxygen consumption rate (OCR) of cardiomyocytes	Zuo et al., 2018
	Total ginsenosides of Radix Ginseng	1	50 mg/L	↑PDH complex (PDC-E2), LDHB, Aldose reductase (ALDR), Glycerol-3-phosphate dehydrogenase [NAD^+^], activating proteins in TCA cycle; activation of PI3K/AKT/eNOS pathways	Wang et al., 2012
*Rhodiola rosea L.*	Salidroside	1	20 and 40 mg/kg/day	↑ATP and glycogen; ↓LDH; ↑p-AMPK, PGC-1α, PPARα; ↓p-NK-κBp65, p-IκBα, p-IKKα, and p-IKKβ	Chang X. Y. et al., 2016
*Ganoderma Lucidum*	Extract	1	100 and 250 mg/kg/day	↑mitochondrial GSH, ↑the activities of mitochondrial enzymes and the activities of respiratory chain complexes such as complexes I, II, III, and IV, ↑MMP, ↓mitochondrial lipid peroxidation, ↓ROS	Sudheesh et al., 2013
	Ganoderma atrum polysaccharide	1	20, 50, and 100 μg/ml	↓ROS, ↑MMP, and ↓ the release of cytochrome c from the mitochondria into cytosol, ↓mitochondria Bax, and ↑ mitochondria Bcl-2	Li et al., 2010
*Gynostemma pentaphyllum (Thunb.) Makino*	Gypenosides	1	50, 100, and 200 mg/kg/day; 5, 10, and 20 μM	↑ATP, ↑COX I, II, IV; ↑Citrate synthase activity, ↑mitochondrial volume; maintaining mitochondrial membrane integrity, ↓ the release of Cyt-c from the mitochondria to the cytosol	Yu et al., 2016
*Panax Notoginseng (Burkill) F.H.Chen*	Notoginsenoside R1	1	5mg/kg/h; 0.1 mM	↑ATP and AMP content, ↓LDH release, ↑ATP5D, ↓ROCK, ↓ p-MYPT1/MYPT1, ↓ p-AMPK	He et al., 2014
*Salvia miltiorrhiza Bunge*	Salvianic acid A	2	10 μM	↑MMP, ↓mPTP opening, ↓c-subunit of ATP synthase; ↓apoptosis	Gao et al., 2017
			5 mg/kg/day	↑ATP/ADP, ATP/AMP, ↑SIRT1, ↑C, ↑Complex I enzyme activity	Yang et al., 2015
	Tanshinone IIA	1	3 μM	↑ATP; ↓mitochondrial superoxide; ↑mitochondrial SOD activity; ↓NO and Ca^2+^ production	Jin and Li, 2013
*Carthamus tinctorius L*	Hydroxysafflor yellow A	2	100 mg/kg; 10 µM	↓ROS, ↓the loss of ΔΨm, ↑PGC-1α, and Nrf2	Chen et al., 2016
			5 µM	↓LDH, ↑mitochondrial ATP, ↓mitochondrial cytochrome c release, weakly p-GSK3-beta, ↑Akt, and HKII	Min and Wei, 2017
*Boswellia serrata Roxb.*	Acetyl-11-keto-β-boswellic acid	1	100 mg/kg; 10 µM	↓CK-MB and LDH, ↓ROS, ↓the loss of ΔΨm, ↑PGC-1α, and Nrf2	Chen et al., 2016
*Cistanche deserticola Ma*	Extract	1	0.5 and 1.0 g/kg/day	↑mitochondrial GSH and MMP, ↓mitochondrial GSSG, and Ca^2+^ content, ↑mitochondrial respiration	Siu and Ko, 2010
	Herba Cistanches fraction	1	1.14 and 3.41 mg/kg/day; 30 ng/ml	↑ATP-GC in mitochondria, ↑mitochondrial GSH status, ↑mitochondrial state 3 and state 4 respiratory	Wong and Ko, 2013
	β-Sitosterol	1	Male, 7 and 70 μg/kg/day; Female, 3.5 and 35 μg/kg/day; 1,3 and 10 μM	↓LDH, ↑cellular glutathione redox cycling in female rats; ↓mitochondrial coupling efficiency, and ↑ mitochondrial state 3 and state 4 respiration	Wong et al., 2014a
*Cynomorium coccineum subsp. songaricum (Rupr.) J.Léonard*	Cynomorii herba fraction	1	Male, 16 or 48 mg/kg/day; Female, 2 or 5 mg/kg/day.	↓LDH, ↑ cardiac mitochondrial ATP-GC and tissue ATP level, ↑the mitochondrial GSH/GSSG ratio, ↑Mitochondrial GR activity	Chen and Ko, 2013
	Ursolic acid	1	Male: 12 or 36 mg/kg/day; Female: 1.5 or 3.5 mg/kg/day	↓LDH, ↑ cardiac mitochondrial ATP-GC and tissue ATP level, ↑the mitochondrial GSH/GSSG ratio, ↑Mitochondrial GR activity	Chen and Ko, 2013
*Coptis chinensis Franch.*	Berberine	1	100 mg/kg/day	↓p-AMPK/AMPK, ↓ADP/ATP, and AMP/ATP in myocardial risk areas; ↑p-AMPK/AMPK, ADP/ATP, and AMP/ATP ratio in non-ischemia area	Chang et al., 2012
*Paeonia lactiflora Pall.*	Crude terpene glycoside	1	300 mg/kg/day; 200 μg/mL	↑ATP and glycogen levels, improve mitochondrial ultrastructure, ↓p-AMPK/AMPK	Ke et al., 2017
*Ginkgo biloba L.*	Extract	2	200 mg/kg/day	restore fatty acid, sphingolipid, phosphoglyceride, glyceride, amino acid, and energy metabolism	Wang et al., 2016b
			0.32 mL/kg/day	uncoupled mitochondrial oxidation from phosphorylation, ↓ the generation of free radicals in the mitochondria, ↓ the degree of respiration stimulation by exogenous cytochrome c.	Bernatoniene et al., 2011
Vegetables, fruits, and medicinal herbs	Luteolin	1	20 μM	↑ATP and CS activity; ↑MMP; ↑Complexs I-V in cardiomyocyte mitochondria	Hu et al., 2016
Vegetables and fruits	Quercetin	2	10 mg/kg/day	↑ATP; ↓myocardial infarct size; ↑mitochondria TBARS and LOOH; ↑mitochondria GPx and GRx; ↑mitochondria enzymes; ↑mitochondria cytochrome-c-oxidase	Punithavathi and Prince, 2010
			10 mg/kg/day	↓CHO, TG, and FFA, phospholipids in serum, ameliorating lipids, lipoproteins, and enzymes of lipid metabolism	Prince and Sathya, 2010
Blueberries, grapes, and cranberries	Resveratrol	2	1 mg/kg/day	↑ATP, PCr, and TAN, ↑Citrate synthase activity, ↑p-AKT, eNOS, and SIRT1	Fourny et al., 2019
			50 mg/kg/day	↑ATP; p-AMPK↑; ↓p-mTOR; resveratrol had no significant effect on SIRT1	Kanamori et al., 2013
*Stephania tetrandra S. Moore*	Tetrandrine	1	50 mg/kg/day; 10 mM	↓LDH, ↑MMP, ↑p-AKT, and GSK-3β, ↓cytosolic translocation of mitochondrial cytochrome c.	Yang et al., 2017b

↑, increase after treatment; ↓, decrease after treatment.

**Table 2:** Metabolic effect and mechanism of Chinese herbal formula in IHD.

Formula	Composition	Frequency (N)	Dosage	Effects/Mechanism of Action	Reference
Buyang Huanwu Decoction	*Astragali Radix*, *Angelica sinensis (Oliv.) Diels*, *Radix Paeoniae Rubra*, *Ligusticum striatum DC.*, *Pheretima*, *Semen Persicae*, and *Carthamus tinctorius L.*	1	25.68, 12.84, and 6.42 g/kg/day	↑Na^+^-K^+^-ATPase activity; ↓ blood glucose	Wang et al., 2011
Shenmai San	*Panax ginseng C.A.Mey*, *Ophiopogon japonicus (Thunb.) Ker Gawl*, and *Schisandra chinensis (Turcz.) Baill*	1	728 mg/kg/day ;25, 100, and 400 µg/mL	↑ATP and MMP, ↓intracellular Ca^2+^ levels, ↑ATPase activity, ↓p-Drp1(Ser616)/Drp1, ↑p-Drp1(Ser637)/Drp1, ↓CaN	Yang et al., 2017d
Qishen granules	*Astragali Radix*, *Radix salvia miltiorrhizae*, *Flos Lonicerae*, *Radix Scrophulariae*, *Radix Aconiti Lateralis Preparata,* and *Radix Glycyrrhizae*.	1	18.66 g/kg/day	↓TC, TG, and LDL-C, mediating FA uptake, transportation into mitochondria and β-oxidation, ↑transcriptional regulators of FA metabolism (PPARα, RXRα, RXRβ, and RXRγ); ↓glycolytic activity (↓PDK4), ↑TCA cycle, ↑SUCLA2, CKMT2, and PGC-1α, ↓UCP2	Gao et al., 2020
Yiqihuoxue Decoction	*Astragali Radix*, *Angelica sinensis (Oliv.) Diels*, *Panax ginseng*, *Ligusticum striatum DC.* and *Panax notoginseng*	1	8.2 g/kg/day	↑ATP and restore mitochondrial structure; ↓ROS; ↑p-AMPK, PGC-1α, Tfam, and NRF1	Li et al., 2018a
Gualou Xiebai Decoction	*Trichosanthis Pericarpium*, *Allium macrostemon Bunge,* and wine	1	4 g/kg/day	↓ADP/ATP and AMP/ATP; ↓TC, TG, HDL-c, and LDL-c, ↑ATP5D, ↓RhoA, and ROCK	Yan et al., 2018
Qishenyiqi capsule	*Astragali Radix*, *Salvia miltiorrhiza*, *Panax notoginseng*, and *Dalbergia odorifera*	4	0.6 mg/kg/day (i.g.)	↑cTnI; ↑ATP/ADP and ATP/AMP; ↑ATP5D, Complex V, and ATP synthase activity	Cui et al., 2018
			0.12, 0.6, and 1.2 g/kg/day	↓ADP/ATP and AMP/ATP; ↑ATP5D, ↓p-MLC, ameliorating mitochondria swelling	Lin et al., 2013
			0.2 g/mL	↑mitochondria ATP; ↑ATP5D; ↓mitochondrial Cyt C	Chen et al., 2015
			100 mg/kg/day	protected the cell nucleus number and mitochondrial mass	Zhang et al., 2018d
Qiliqiangxin capsule	*Panax ginseng*, *Astragali Radix*, *Aconiti lateralis radix preparata*, *Semen Lepidii Apetali*, *Salvia miltiorrhiza Bunge*, *alismatis rhizoma*, *cinnamomi ramulus*, *Polygonatum odoratum (Mill.) Druce*, *Periploca sepium Bunge*, *Carthamus tinctorius L.*, and *citri reticulatae pericarpium*	3	0.5 g/kg/day	↑9 energy metabolism-related genes (Cd36, Fatp, Pdk4, Acadm, Acadl, Acadvl, Cpt1a, Cpt1b, and Cpt2), ↑ PPARγ	Shen et al., 2017
			0.5 mg/mL	↑ATP and MMP, ↓ROS, ↑GLUT1, HK2, PFK1, PKM2, LDHA, HIF-1α, and VEGF; ↓pAMPK/AMPK	Wang et al., 2018a
			0.25, 0.5, and 1.0 g/kd/d; 90 µg/mL	↓LDH, ROS, and mPTP opening, ↑MMP, ↑p-AKT/AKT, p-GSK3β/GSK3β, ↑p-Drp1(Ser637)/Drp1	Zhao et al., 2019
Compound danshen dripping pills	*Salvia miltiorrhiza Bunge*, *Radix Notoginseng* and *Borneolum*	1	167 mg/kg/day	↓CK and LDH, ↓PCr/ATP, and ADP/AT; ↑the flux of pyruvate into the TCA cycle; ↑ fatty acids β-oxidation and 3-hydroxybutyrate	Guo et al., 2015
DanQi pill	*Salvia miltiorrhiza Bunge* and *Panax notoginseng (Burkill) F.H.Chen*	6	1.5 mg/kg/day	↑LPL; ↓cytochrome P450; ↑PPARα, PPARγ, CPT-1A, FABP4, RXRA, and PGC-1α;	Wang et al., 2016a
			1.5 mg/kg/day	↑ApoA-1, FABP, CPT-1A, PPARα, and CD36;	Chang H. et al., 2016
			1.5 mg/kg/day	↓TG, LDL, Apo-B, and HMGCR; ↑PPARα, RXRs, FATP, and CPT1	Wang et al., 2015a
			1.5 mg/kg/day	↑ATP; ↑PGC-1α, SIRT1, AMPK, MFN1; ↑SOD2	Meng et al., 2019
			1.5 mg/kg/day	↑ATP, ADP, and EC, ↓AMP and energy charge↑; ↓FFA; ↑lipid metabolism enzymes (ACADL and SCP2); PPARγ↑; ↓Glucose and Lactate, ↑Glycogen; ↑GLUT4, PFK, and GS; ↓^18^F-FDG uptake SUV, ↓GSK-3β, and p-GS	Zhang et al., 2018c
			1.5 g/kg/day; 400 μg/mL	↑PPARα, CPT1A, and CD36	Jiao et al., 2018
Yangxinshi tablet	*Astragali Radix*, *Codonopsis pilosula*, *Salvia miltiorrhiza Bunge*, *Pueraria lobata (Willd.) ohwi*, *Epimedium brevicornu Maxim, Crataegus pinnatifida Bunge*, *Rehmannia glutinosa (Gaertn.) DC.*, *Angelica sinensis*, *Coptis chinensis Franch.*, *Corydalis yanhusuo*, *Ganoderma lucidum*, *Panax ginseng C.A.Mey.* and *Glycyrrhiza uralensis Fisch. ex DC.*	1	500 and 1000 mg/kg/day	↑ATP, ↑p-AMPK/AMPK, PGC-1α, GLUT4, HIF-1α, ↑MMP, mitochondrial complex I activity	Wu et al., 2020b
Shengmai injection	*Panax ginseng C.A.Mey.* and *Ophiopogon japonicus (Thunb.) Ker Gawl.*	2	10 mL/kg; 20 μL/mL	↑MMP and ATP, ↑ATP5D, NDUFB10, MDH1, and TNNC1, ↑OCR, ↑ MDH1, and ATP5F1	Wang et al., 2018b
			0.38 mL/kg; 1, 2.5, 5 μL/mL	↓mitochondrial mass and cytosolic Ca^2+^, ↑MMP, ↓mPTP opening, ↑MFN1, MFN2, and OPA, ↓DRP and FIS	Yu et al., 2019
Total Salvianolic Acid injection	Active components of *Salvia miltiorrhiza Bunge*	1	8 mg/kg/h; 0.013 mg/mL	↑ATP, ↑ATP5D, SIRT1, SIRT3, and ↑NDUFA10, ↑SDHA, and restoring mitochondrial respiratory chain complexes activity.	Huang et al., 2019
Xuesaitong injection	*Panax Notoginseng* saponins	1	80 mg/kg/day; 100, 200, 400 μg/mL	↑activity of PDH, ↑intracellular content of acetyl-CoA, and ATP, ↑PDHA1, ATP5A.	Zhao et al., 2017
YiQiFuMai powder injection	*Panax ginseng* C.A.Mey., *Ophiopogon japonicus (Thunb.) Ker Gawl* and *Schisandra chinensis (Turcz.) Baill*	2	0.13, 0.26, and 0.53 g/kg/day; 25–800 μg/mL	Improving mitochondrial morphology, ↑ Δψm, ↑Mfn2, ↓ p-Drp1/Drp1, ↓NADPH, NOX2, and NOX4, ↓ROS.	Zhang et al., 2019
			1.06 g/kg/day; 25–400 μg/mL	↑ATP and MMP, ↑p-AMPKα/AMPKα	Li et al., 2016a

↑, increase after treatment; ↓, decrease after treatment.

### Metabolic Effects and Mechanisms of Herbs and Major Bioactive Components

#### Invigorating and Replenishing Qi

##### Astragalus mongholicus Bunge (Astragali Radix)

*Astragalus mongholicus Bunge* (*Astragalus membranaceus*, AM), also known as Huang-qi in China, is considered as one of the major replenishing Qi medicines. Classified as a top-grade herb in “Shen Nong Ben Cao Jing”, *Astragalus mongholicus Bunge* is widely used for the treatment of cardiovascular diseases (Ma et al., 2013). Recent studies have focused on its cardioprotective effects, especially those related to improving energy metabolism. Astragali Radix extract (ARE) exerts a cardioprotective effect against LAD ligation-induced myocardial infarction by rectifying the levels of FFA, pyruvic acid (PA) and lactic acid (LA) in serum and myocardial tissue, thereby producing more energy (Jin et al., 2014). Astragalosides are roughly extracted from Astragali Radix. Astragalosides (5 mg/kg/day, i.p.) showed protective effects by rebalancing intracellular Ca^2+^ homeostasis and regulating energy metabolism in Iso-induced myocardial ischemic injury. However, the mechanism of Astragalosides has yet to be reported (Chen et al., 2006). Astragalosdie IV (AS-IV), a major bioactive component of the astragalosides, has been reported to improve cardiac dysfunction and modulate energy metabolism in MI rat model. The metabolic mechanism may be mediated *via* the promotion of Complex V and ATP synthase delta-subunit (ATP5D) expression (Cui et al., 2018). Another trial identified the metabolic roles of AS-IV in myocardial ischemia and ischemia/reperfusion injury. AS-IV also enhanced the expression of ATP5D and Complex V (Tu et al., 2013). These results indicate that AS-IV may regulate energy metabolism through mitochondrial respiration. Besides, AS-IV can modulate energy biosynthesis. Zhang et al. (2015) found that AS-IV improved the cardiac hemodynamics, mediated energy biosynthesis, and upregulated ATP5D and PGC-1α expression in Iso-induced cardiac injury. In neonatal rat ventricular myocytes (NRVMs), the cardioprotective mechanism of AS-IV may be mediated through regulating nuclear factor NF-κB/PGC-1α signaling (Zhang et al., 2015). Glycogen synthase kinase-3β (GSK-3β), a serine/threonine protein kinase, interacts with mitochondrial proteins such as PI3K-Akt, PGC-1α, and subunits of mPTP, which plays an essential role in relating to mitochondrial biogenesis, mitochondrial permeability, and glycogen metabolism (Yang et al., 2017a). Formononetin is the main isoflavonoids compound of Radix Astragali. Formononetin enhanced GSK-3β and Akt phosphorylation in H9c2 cells during oxygen glucose deprivation (OGD) and reoxygenation, thereby reducing GSK-3β activity towards mPTP opening (Cheng et al., 2016). Kaempferol, a natural flavonoid, exists in *Astragalus mongholicus Bunge* and *Panax ginseng C.A.Mey.* Kaempferol showed cardioprotective effects *via* mitochondrial pathway against ischemia/reperfusion injury in NRVMs. The cardioprotective mechanisms may be mediated by SIRT1 (Guo et al., 2015). Astragalus polysaccharides (AP) had ability to improve cardiac energy biosynthesis and prevent Iso-induced cardiac ischemic injury by regulating tumor necrosis factor TNF-α/PGC-1α signaling-mediated energy biosynthesis, both *in vivo* and *in vitro*. Among them, ATP5D, PGC-1α, and pyruvate dehydrogenase kinase isoform 4 (PDK4) all increased, which means that AP may be related to energy metabolism (Luan et al., 2015).

##### Panax ginseng *C.A.Mey.* (RG)

*Panax ginseng* C.A.Mey.*(Radix ginseng)*, also known as Ren Shen, is well known for its “Qi-Replenishing” effect in TCM and is listed as a top-grade herb in “Shen Nong Ben Cao Jing”. In the last decade, the representative active ingredients of Radix ginseng (including Ginsenoside Rb1, Ginsenoside Rd, Ginsenoside Rg1, Ginsenoside Rg5, Panax ginseng Polysaccharide, and total ginsenosides) have been demonstrated to exert significant effects on energy metabolism. Ginsenoside Rb1(Rb1), a major effective ingredient of *Panax ginseng*, has been shown to modulate energy metabolism in myocardial ischemia and reperfusion injury, hypertrophy, and even HF (Zheng et al., 2017). In rat models of myocardial infarction, Rb1 could increase the expression of the mitochondrial ATP5D and complex V (Cui et al., 2018). In ischemia/reperfusion injury, Rb1 reduced the infarction sizes, inhibited of mPTP opening, restored of the MMP, and upregulated the p-AKT and p-GSK-3β expression. These results indicate that the protective effects of Rb1 against I/R-induced myocardial injury may be associated with the protection of mitochondrial function (Li et al., 2016b). Similarly, Rb1 could protect cardiac myocytes and modulate energy metabolism against I/R-induced myocardial injury *via* RhoA signaling pathway (Cui et al., 2017). Ginsenoside Rd (Rd) is another biologically active extract from *Panax ginseng* C.A.Mey. Wang et al. (2013) found that Rd exerted cardioprotective effects by stabilizing the MMP and attenuating the release of mitochondrial cytochrome c in myocardial ischemia/reperfusion injury. As a major compound of *Radix ginseng*, Ginsenoside Rg1 (Rg1) modulated energy metabolism in ischemia/reperfusion injury by enhancing ATP content and the activity of mitochondria respiratory chain complexes, which might partially be related to its binding to RhoA and consequent the inhibition of RhoA/ROCK pathway (Li et al., 2018b). In vitro, Rg1 treatment (12.5 μM) exerted a cardioprotective effect through regulating mitochondrial dynamics, and achieved by moderating glutamate dehydrogenase (GDH) and MFN2 dysregulation. However, Rg1 had no significant effect on MFN1, OPA1, and Drp1 (Dong et al., 2016). Mitochondrial hexokinase-II (HK-II), as a key molecule in glycolysis, has ability to keep the mitochondrial integrity and prevent mitochondrial death (Roberts and Miyamoto, 2015). Ginsenoside Rg5 (Rg5) ameliorated the iso-induced ischemic myocardium injury through inhibiting fatty acid oxidation and regulating mitochondrial dynamics imbalance. Rg5 may improve mitochondrial dysfunction through regulating mitochondrial HK-II binding and reducing Drp1 recruitment to mitochondria *via* Akt activation (Yang et al., 2017c). Panax ginseng Polysaccharide (PGP) had cardioprotective effects and protected mitochondrial function in myocardial I/R injury. In vitro, PGP reduced the release of mitochondrial cytochrome c, maintained the MMP, and restored mitochondrial respiration (Zuo et al., 2018). Total ginsenosides (TGS) of RG have been reported to enhance energy metabolism by increasing glucose metabolism and activating TCA cycle-related protein expression in ischemic rat myocardium (Wang et al., 2012).

##### Rhodiola rosea L. (RR)

*Rhodiola rosea L.*, a well-known plant in Tibet, has been demonstrated to treat a diverse range of cardiovascular conditions, including IHD, arrhythmia, and angina pectoris (Yu et al., 2014; Liu et al., 2016). Salidroside (SAL) is the main component extracted and purified from Rhodiola. Chang et al. (2016) reported that SAL had cardioprotective effects by regulating energy metabolism in coronary artery occlusion-induced myocardial injury. SAL enhanced the ATP and glycogen content through AMPK/PGC-1α axis and AMPK/NF-κB signaling pathways (Chang X. Y. et al., 2016).

##### Ganoderma Lucidum (GL)

*Ganoderma Lucidum (Reishi mushroom)*, popularly known as Lingzhi in Asian countries, has antioxidative and cardioprotective effects. *Ganoderma Lucidum* extract ameliorated myocardial ischemic injury by improving mitochondrial dysfunction in iso-induced myocardial infarction rats. The mechanism may be related to the activities of the enzymes of TCA cycle and mitochondrial respiratory chain complexes such as complexes I, II, III, and IV (Sudheesh et al., 2013). Ganoderma atrum polysaccharide (PSG-1) is regarded as a major bioactive ingredient in *Ganoderma Lucidum*. Li et al. (2010) reported that PSG-1 protected cardiomyocytes by mitochondrial pathway in hypoxia/reoxygenation-induced NRVMs injury. PSG-1 reduced the release of cytochrome c from the mitochondria into cytosol and enhanced MMP levels (Li et al., 2010).

##### Gynostemma pentaphyllum *(Thunb.) Makino (GPM)*

As one of replenishing Qi medicines, *Gynostemma pentaphyllum* (Thunb.) Makino exerts anti-hypertensive, anti-hyperlipidemia, anti-inflammation, and anti-aging effects (Zhang et al., 2018a). Gypenosides (GP) are the major saponins of *Gynostemma pentaphyllum*, which possess cardioprotective effects in myocardial infarction rats. Yu et al. (2016) found that GP significantly reduced myocardial infarct size and protected mitochondrial function in myocardial ischemia-reperfusion injury. GP enhanced the levels of ATP, regulated enzymatic activities of mitochondrial respiration chain, and maintained the mitochondrial membrane integrity (Yu et al., 2016).

#### Replenishing and Activating Blood

##### Panax notoginseng (Burkill) *F.H.Chen (PNG)*

*Panax notoginseng (Burkill)* F.H.Chen, known as San qi, San chi, and/or Tian qi in China, is a blood-replenishing and stasis-resolving TCM herb widely used in the treatment of cardiovascular diseases. *Panax Notoginseng* can reduce MI size and CK levels in rat models of myocardial ischemia (Han et al., 2013). There are three main saponins of *Panax Notoginseng*, including Rg1, Rb1, and Notoginsenoside R1 (R1). R1 is the major effective component of *Panax notoginseng* and exerts significant cardioprotective effects by preventing the dysregulation of energy metabolism. The energy metabolism-associated modulatory mechanism of R1 may be mediated through inhibiting the activity of ROCK, and elevating ATP5D expression and ATP content in ischemia/reperfusion-induced myocardial injury (He et al., 2014).

##### Salvia miltiorrhiza Bunge (SM)

*Salvia miltiorrhiza Bunge* (also called Danshen), another blood-replenishing and stasis-resolving TCM herb, has shown therapeutic promise for multiple cardiovascular diseases. Salvianic acid A (Danshensu) is one of the major water-soluble compounds derived from *Salvia miltiorrhiza* and has been reported to alleviate cardiac I/R injury by inhibiting mPTP opening and expression of the ATP synthase c-subunit (Gao et al., 2017). 3, 4-Dihydroxylphenyl lactic acid (DLA) is another name for Salvianic acid A, which has been demonstrated to reduce infarction size and enhance mitochondrial aerobic respiration in myocardial I/R injury. NADH dehydrogenase 1 alpha subcomplex 10 (NDUFA10) belongs to one of the subunits of mitochondrial Complex I. DLA may regulate the mitochondrial respiration *via* enhancing the NDUFA10 and SIRT1 expression (Yang et al., 2015). Sirtuin3 (SIRT3), another NAD^+^-dependent deacetylases in addition to SIRT1, modulates the succinate dehydrogenase complex, subunit A, flavoprotein variant (SDHA). Total Salvianolic Acid Injection (TSI) is a preparation of an active compound extracted from *Salvia miltiorrhiza Bunge*. TSI had significant cardioprotective effects by regulating mitochondrial respiratory chain in ischemia/reperfusion-induced myocardial injury. TSI reduced infarction size and enhanced NDUFA10 and SDHA protein expression *via* the activation of SIRT1 and SIRT3 (Huang et al., 2019). Tanshinone IIA (TIIA), another monomer isolated from the *Salvia miltiorrhiza*, has been reported to protect cardiac function against MI and I/R injury. Jin and Li (2013) found that TIIA protected mitochondrial function in hypoxia-induced H9c2 cells injury, which might involve the regulation of mitochondrial ROS generation, ATP content, and intracellular nitric oxide (NO) levels (Jin and Li, 2013). However, the mechanism of TIIA has yet to be revealed.

##### Carthamus tinctorius L

*Carthamus tinctorius L* (CTL) is widely used in Asian countries, which exerts significant capacity of anticoagulation, increasing coronary blood flow, and treating ischemic cardiovascular diseases (Zhou et al., 2014). Hydroxysafflor Yellow A (HSYA) is a major representative ingredient of *Carthamus tinctorius L*. HSYA significantly reduced the blood CK-MB and LDH levels, mitochondrial ROS accumulation, and the loss of MMP in iso-induced myocardial ischemic injury. In vitro, HSYA markedly increased the PGC-1α and Nrf2 protein expression in H9c2 cells subjected to OGD injury (Chen et al., 2016). HKII, located on the outer mitochondrial membrane, regulate cardiac mitochondrial function and cellular glucose metabolism (Roberts and Miyamoto, 2015). Min and Wei (2017) dived more deeply into the cardioprotective mechanism of HSYA in ischemia-reperfusion myocardial injury. They found that HSYA exerted cardioprotective effects by restoring mitochondrial energy metabolism. This mechanism of HSYA may be mediated *via* Akt/HKII independent of ERK/GSK-β signal pathway. Taken together, these results suggest that HSYA effectively improve myocardial injury in pre-clinical models of myocardial ischemia.

##### Boswellia serrata Roxb.

*Boswellia serrata Roxb.* (BSR) is considered as one of a major promoting blood circulation for relieving pain TCM herb. The combination of *Carthamus tinctorius L* and *Boswellia serrata Roxb.* is widely used for treating ischemic heart disease (Chen et al., 2016). Acetyl-11-keto-β-boswellic acid (AKBA) is recognized as the main component of *Boswellia serrata Roxb.*, which has similar cardioprotective actions as Hydroxysafflor Yellow A in H9c2 cells subjected to OGD injury. AKBA effectively improved mitochondrial membrane potential levels and increased the PGC-1α and Nrf2 protein expression, suggesting that the beneficial role of AKBA in OGD-induced myocardial ischemic injury can be attributed to the improvement of mitochondrial biogenesis (Chen et al., 2016).

#### Invigorating and Replenishing Yang

##### Cistanche deserticola Ma

*Cistanche deserticola Ma*, also known as *Herba Cistanche* (HC), is a “Yang-invigorating” Chinese tonic herb. *Herba Cistanche* extract protected against myocardial ischemia/reperfusion injury by enhancing mitochondrial ATP generation. Glutathione (GSH) is the first defense line against oxidative damage. Herba Cistanche extract may enhance mitochondrial respiration through increasing mitochondrial GSH levels, decreasing mitochondrial oxidized glutathione (GSSG), improving MMP, and reducing mitochondrial Ca^2+^ levels (Siu and Ko, 2010). Another study reported the cardioprotective effect of *Herba Cistanches* fraction (HCF1) *in vitro* and *in vivo*. In vitro, HCF1 at 30 ng/mL significantly enhanced mitochondrial ATP generation capacity (ATP-GC), mitochondrial ROS production and mitochondrial respiration. In vivo, HCF1 significantly enhanced mitochondrial GSH status and enhanced ATP-GC content in I/R adult female rats. Surprisingly, they found that low-dose HCF1 significantly reduced tissue ATP levels in non-I/R rats, whereas the depletion of tissue ATP level by HCF-1 was reduced in I/R rats. This phenomenon in non-I/R rats may be related to HCF-induced sustained mitochondrial uncoupling, while in I/R rats it may be related to the increase in ATP-GC by HCF-1 (Wong and Ko, 2013). β-sitosterol (BS), as a hydrophobic compound of HCF1, reduced LDH levels and increased the cellular glutathione redox cycling in myocardial I/R female rats. But BS had no significant effects on mitochondrial ATP-GC in male or female rat myocardium (Wong et al., 2014). Therefore, the effects of mitochondrial ATP-GC in *Cistanche deserticola Ma* extract may depend on other compounds, which need to be further studied.

##### Cynomorium coccineum subsp. songaricum (Rupr.) J.Léonard

*Cynomorium coccineum* subsp*. songaricum (Rupr.) J. Léonard* (also called *Cynomorii herba*) (CCS), an another “Yang-invigorating” Chinese tonic herb, is found to enhance mitochondrial ATP-GC in H9c2 cells. Chen and Ko (2013) isolated bioactivate fraction (HCY2) and ursolic acid (UA) from *cynomorii herba*. They demonstrated that HCY2 and UA could protect myocardial ischemia/reoxygenation in male and female rats. HCY2 and UA may protect mitochondrial function through reducing LDH level, enhancing cardiac tissue ATP and mitochondrial ATP-GC levels, and upregulating the mitochondrial GSH/GSSG ratio and glutathione reductase (GR) activity.

#### Other Extractive Compounds of Chinese Herbal Medicines

##### Berberine

Berberine, an isoquinoline alkaloid isolated from *Coptis chinensis Franch.*, is widely used in treatment of diarrhea in Asian countries. Wang et al. (2015b) reported that berberine (200 mg/kg/day) exerted cardioprotective effects by attenuating myocardial apoptosis and improving mitochondrial dysfunction in myocardial ischemia/reperfusion. However, the metabolic mechanism of berberine remains unclear. Berberine treatment (100 mg/kg/day, i.g.) improved cardiac function and reduced infarction size. Berberine may have cardioprotective effects through regulating AMPK phosphorylation in ischemia-reperfusion myocardial injury. Interestingly, they found that berberine downregulated p-AMPK expression, and decreased the ADP/ATP and AMP/ATP ratio in peri-infarct areas. In contrast, berberine upregulated p-AMPK expression, and increased the ADP/ATP and AMP/ATP ratio in non-ischemia areas. However, this phenomenon has yet to be revealed (Chang et al., 2012).

##### Crude *Terpene Glycosides*

Crude terpene glycosides (CS-TG), as the major active components in *Paeonia lactiflora Pall.*, include paeoniflorin, albiflorin, and enzoylpaeoniflorin. Crude terpene glycosides have been shown to attenuate cardiac hypertrophy, inhibit cardiomyocyte apoptosis and promote blood flow (Ke et al., 2017). Ke et al. (2017) conducted the myocardial ischemia model in rats fed with 300 mg/kg/day CS-TG. CS-TG significantly decreased CK and LDH levels in serum and improved energy metabolism. Rats treated with CS-TG improved energy metabolism in ISO-induced myocardial ischemic injury, which might be related to increasing the ATP and glycogen content, protecting mitochondrial ultrastructure and decreasing p-AMPK expression (Ke et al., 2017). In contrast, many studies indicated that the activation of AMPK phosphorylation promotes energy metabolism by regulating glucose and lipid metabolism (Luiken et al., 2003; Russell et al., 2004; Qi and Young, 2015). AMPK activation also promotes mitochondrial fission *via* MFF phosphorylation (Garcia and Shaw, 2017). The inconsistency between the upregulation and downregulation on AMPK phosphorylation during ischemia may depend upon the cell type, myocardial ischemic model, pathophysiological setting and ischemic duration. Besides, inhibition of AMPK is likely to involve other mechanisms such as myocardial acidosis and apoptosis.

##### Ginkgo biloba L. *Extract*

*Ginkgo biloba L*. (GBE) extract is one of the most commonly used herbs and exhibits multiple pharmacological activities. GBE has been widely used for the treatment of cardiovascular diseases.

A study by Wang et al. (2016b) reported that GBE (200 mg/kg/day) pretreatment could significantly restore fatty acid, glyceride, and amino acid levels, thereby exerting cardioprotective effects in ISO-induced myocardial ischemia in rats. Another study found that GBE treatment uncoupled mitochondrial oxidative phosphorylation and reduced the mitochondrial free radicals in ischemic rat heart for 10 and 18 days (Bernatoniene et al., 2011).

##### Luteolin

Luteolin is a polyphenolic compound derived from vegetables, fruits, and medicinal herbs. In vivo, Hu et al. (2016) reported that Luteolin could improve cardiac dysfunction in wild-type mice after myocardial infarction. Meanwhile, luteolin treatment was shown to enhance MMP levels, ATP content, citrate synthase (CS) activity, and the activities of complexes I-V induced by hypoxia in neonatal mice ventricular cardiomyocytes. The cardioprotective effects of luteolin associated with improvement in mitochondrial biogenesis may be exerted through inhibition of mammalian sterile 20-like kinase 1 (Mst1) expression.

##### Quercetin

Quercetin, a flavone used as a food supplement, exerts powerful antioxidant effects. Punithavathi and Prince (2010) demonstrated that quercetin reduced myocardial infarct size and prevented the mitochondrial dysfunction in isoproterenol-treated myocardial infarcted rats. Another study also found quercetin (10 mg/kg) pre-treatment elicited cardioprotective effects, including ameliorating lipids accumulation and altering the levels of lipoproteins and enzymes involved in lipid metabolism in isoproterenol-treated myocardial infarcted rats (Prince and Sathya, 2010). However, the protective mechanism remains unclear.

##### Resveratrol

Resveratrol is a natural polyphenol from many plant-based foods, including blueberries, grapes, and cranberries. Multiple studies have reported that resveratrol exerts cardioprotective effects (Kanamori et al., 2013; Sung et al., 2015; Fourny et al., 2019). Kanamori et al. (2013) investigated the effects of resveratrol in myocardial infarction mice and found that resveratrol could improve myocardial energy status through enhancing the ATP content and increasing the p-AMPK activation. Furthermore, Fourny et al. (2019) also focused on the cardioprotective effects of resveratrol against I/R injury, which is related to improving mitochondrial dysfunction. They found that the underlying mechanism may be associated with increased expression of p-AKT, eNOS, and SIRT1, resulting in improved energy metabolism.

##### Tetrandrine

Tetrandrine (TTD), a bisbenzylisoquinoline alkaloid isolated from *Stephania tetrandra S. Moore*, has been shown cardioprotective effects on myocardial fibrosis and myocardial infarction (Teng et al., 2015). In vivo, tetrandrine (50 mg/kg) pretreatment significantly improved cardiac function, reduced infarct size and decreased blood LDH levels in myocardial ischemia and reperfusion injury. In neonatal rat cardiomyocytes, tetrandrine treatment (10 mM) significantly reduced mitochondrial ROS accumulation, stabilized the MMP, attenuated mitochondrial cytochrome c release, and enhanced p-AKT and p-GSK-3β protein expression. This phenomenon may be related to mitochondrial function (Yang et al., 2017b).

### Metabolic Effects of Chinese Herbal Formulas and the Associated Mechanisms

In addition to herbs and the major bioactive components described above, we also summarize the metabolic effects and associated mechanisms of Chinese herbal formulas in IHD. Chinese herbal formulas (including decoctions, Chinese patent drugs, and injections), which refer to the combination of specific herbs based on TCM theory, are widely used in Chinese clinical practice. However, the research of formulas has faced numerous obstacles and challenges because of the complexity associated with pharmacological properties of multi-herb, multi-component, and multi-target. In recent years, researchers begin to address the complexity of biology in formulas from a systems perspective using the modern science and advanced technologies such as quality control, metabonomics, and molecular biology. The new technologies are important for ensuring standardization and industrialization of CHMs and identifying the optimal treatment for cardiovascular diseases.

#### Decoctions

##### Buyang Huanwu Decoction

Buyang Huanwu Decoction (BYHWD), a classic TCM formula of qi-replenishing and stasis-eliminating method, is containing *Astragalus mongholicus Bunge*, *Angelica sinensis (Oliv.) Diels*, *Radix Paeoniae Rubra*, *Ligusticum striatum DC*, *Pheretima*, *Semen Persicae*, and *Carthamus tinctorius L*. BYHWD may relieve MI injury through regulating energy metabolism in rats with coronary heart disease (Wang et al., 2011). However, the metabolic mechanism of BYHWD has yet to be revealed.

##### Shengmai San

Shengmai San (SMS), a well-known TCM prescription comprising *Panax ginseng* C.A.Mey, *Ophiopogon japonicus* (Thunb.) Ker Gawl, and *Schisandra chinensis* (Turcz.) Baill, is widely used to treat coronary artery disease, angina pectoris, and HF in clinical practice. SMS water extract significantly improved cardiac function, increased ATPase activity during 3 weeks in MI-induced heart failure mice model. In vitro, SMS (400 µg/mL) could improve mitochondrial function by enhancing MMP and ATP levels. Besides, SMS inhibited phosphorylation of Drp1 at Ser 616 and increased phosphorylation of Drp1 at Ser 637 in OGD-induced cardiomyocytes injury (Yang et al., 2017d). Drp1 has two major phosphorylation sites. Phosphorylation of Drp1 at Ser616 leads to mitochondrial fission, while Drp1 phosphorylation at Ser637 inhibits mitochondrial fission and induces mitochondrial fusion and elongation (Willems et al., 2015). These results indicate that the mechanism of SMS may be associated with inhibiting mitochondrial fission through Drp1 signaling pathways.

##### Qishen Granule

Qishen granule (QSG) consists of 6 Chinese herbs, which has been applied to treat cardiovascular diseases for many years in clinic (Wang et al., 2017). QSG elicited significant cardioprotective effects by regulating lipid and glucose metabolism in MI rat model. 28 days after MI, QSG improved cardiac functions and attenuated cardiac remodeling. On the one hand, QSG could regulate the transcription of fatty acid metabolism through PPARα-RXRs pathway. On the other hand, QSG could regulate glucose metabolism through inhibiting glycolysis uncoupling from glucose oxidation. What is more, QSG also facilitated TAC and protected mitochondrial function in HF rats (Gao et al., 2020).

##### Yiqihuoxue Decoction

Yiqihuoxue Decoction (YQHX) is designed based on the Danggui Buxue decoction (DBD) TCM formula, which is a recognized treatment for IHD with Qi deficiency and blood stasis syndrome. Li et al. (2018a) reported that a 28-day administration of YQHX, a formulation containing *Astragalus membranaceus*, *Angelica sinensis (Oliv.) Diels*, *Panax ginseng*, *Ligusticum striatum DC.,* and *Panax notoginseng*, could significantly improve cardiac function and mitochondrial function in myocardial ischemic rats with LAD surgery. YQHX treatment significantly increased PGC-1α expression, improved the mitochondrial ultrastructure, and increased mitochondrial ATP content. In vitro, YQHX largely reduced LDH and ROS levels, restored the mitochondrial morphology, and increased MMP. Meanwhile, YQHX upregulated PGC-1α and NRF-1 protein expression through the activation of p-AMPK phosphorylation induced by ischemia/hypoxia-induced H9c2 cells injury. Among them, AMPK, PGC-1α, NRF-1, and Tfam are all increased which means that the cardioprotective effects of YQHX may be related to improving mitochondrial dysfunction.

##### Gualou Xiebai Decoction

Gualou Xiebai Decoction (GLXB), a classic TCM prescription, is widely used for the treatment of cardiac heart diseases. GLXB is composed of *Trichosanthis Pericarpium*, *Allium macrostemon Bunge* and wine, has been proposed in AD 200–205 by famous doctor Zhang Zhong-Jing. Rats treated with GLXB exhibited a significant reduction in myocardial infarct size, as well as improved cardiac function and myocardial structure following myocardial I/R injury, which was likely achieved through the modulation of energy metabolism *via* the inhibition of the RhoA/ROCK signaling pathway (Yan et al., 2018).

#### Chinese Patent Drugs

##### QishenYiqi Capsule

Qishen Yiqi capsule (QSYQ), a clinically used formula consisting of extracts from *Astragalus membranaceus*, *Salvia miltiorrhiza Bunge*, *Panax notoginseng,* and *Dalbergia odorifera*, has been approved for clinical use in China and is widely used to treat cardiovascular diseases such as IHD, angina pectoris, and ischemic HF (Jianxin et al., 2016; Zhang et al., 2018b). Recent pharmacological studies showed that QSYQ could modulate energy metabolism and improve cardiac function in ischemic rats with LAD coronary artery ligation (Cui et al., 2018; Zhang et al., 2018d). Zhang et al. (2018d) identified 24 chemical ingredients in QSYQ *via* UPLC-Q-TOP/MS in the negative and positive modes. QSYQ treatment could alleviate mitochondrial dysfunction and protect nuclei number and mitochondrial mass against hypoxia/ischemia-induced injury, however, the metabolic mechanism has yet to be revealed. Similarly, QSYQ has been shown to regulate energy metabolism in a rat model of cardiac I/R injury (Lin et al., 2013; Chen et al., 2015).

##### Qiliqiangxin Capsule

Qiliqiangxin capsule (QLQX) is an 11-herb Chinese medication widely used for treating myocardial infarction and even congestive heart failure in clinical practice. In ovariectomized mice, QLQX (0.5 g/kg) treatment significantly attenuated cardiac remodeling and facilitated energy metabolism after myocardial infarction by upregulating the expression of lipid metabolism-related genes and activation of PPARγ (Shen et al., 2017). In rat primary cardiac microvascular endothelial cells (CMECs) subjected to hypoxia, QLQX was found to improve glucose utilization and protect CMECs against hypoxia-induced injury by promoting hypoxia-inducible factor 1-alpha (HIF-1α)-dependent glycolysis (Wang et al., 2018a). Zhao et al. (2019) conducted the myocardial infarction surgery in male SD rats fed with (0.25, 0.5, and 1.0 g/kg/day) QLQX. 4 weeks after myocardial infarction, QLQX treatment protected cardiac function, ameliorated mitochondria-dependent apoptosis, and enhanced p-AKT and p-GSK3β expression. In addition, QLQX also regulated mitochondrial fission, reduced mPTP opening and enhanced MMP levels in oxidative stress-induced cardiomyocytes injury. Taken together, these findings indicate that QLQX may regulate energy metabolism by increasing lipid metabolism, improving glucose utilization, and regulating mitochondrial fission.

##### Compound Danshen Dripping Pill

Compound Danshen dripping pill (CDDP) consists of *Radix Salvia miltiorrhiza*, *Radix Notoginseng,* and *Borneolum*, which is widely used for the treating ischemic heart diseases. Guo et al. (2016) generated a rat model of acute myocardial ischemia induced by isoproterenol, and found that CDDP pretreatment could increase ATP production and modulate metabolomic patterns in ischemic rat myocardium through promoting a metabolic shift toward fatty acids metabolism.

##### DanQi Pill

DanQi pill (DQP) is composed of two herbs, namely, *Salvia Miltiorrhiza* and *Panax Notoginseng*. The formulation is listed in Chinese Pharmacopoeia of 2010 and is widely used for the clinical treatment of IHD. Recent pharmacological studies showed that DQP treatment could significantly improve cardiac function and modulate lipid metabolism in rat models of MI (Wang et al., 2015a; Chang H. et al., 2016; Wang et al., 2016a; Jiao et al., 2018), as well as promote a significant increase in the expression of CPT-1A, CD36, and PPARα. Among them, CPT-1A, CD36, and PPARα expressions are all increased which means that the metabolic mechanism of DQP may be associated with lipid metabolism. Zhang et al. (2018c) conducted HF after MI rat models and oxygen-glucose deprivation-reperfusion (OGD/R)-induced H9c2 cell injury models. They found that DQP had similar actions as a selective PPARγ activator (Rosiglitazone), which rescued cardiac function, and regulated key factors in lipid and glucose metabolism in MI-induced HF rat model through the PPARγ pathway. To further certain the metabolic mechanism of DQP on PPARγ, H9c2 cells were treated with/without PPARγ inhibitor (T0070907) and DQP. They found that the increase of ATP content and PPARγ expression of DQP could be inhibited by T0070907 in OGD/R-induced H9c2 cell injury. Besides, DQP has also been suggested to regulate energy metabolism in rat ischemic myocardium through the AMPK/SIRT1-PGC-1α signaling pathway (Meng et al., 2019).

##### Yangxinshi Tablet

Yangxinshi tablet (YXS) is composed of 13 herbs, which has been widely used to prevent and treat chest tightness, angina pectoris, and coronary heart disease. It is widely used in replenishing Qi, activating blood circulation and resolving blood stasis in clinic. Zhang et al. (2018b) found 25 metabolites from metabolic profiles in ischemia-reperfusion injury. The metabolites were mainly involved in energy metabolism, fatty acid metabolism, and amino acid metabolism. However, the mechanism of YXS needs to be further explored. Another study dived more deeply into the cardioprotective mechanism of YXS. YXS treatment significantly decreased infarct size, protected cardiac function, and improved energy metabolism in rats with chronic ischemic heart failure. YXS improved energy metabolism through increasing p-AMPK, PGC-1α, GLUT4, and HIF-1α expression (Wu et al., 2020b).

#### Injections

##### Shengmai Injection

Shengmai injection (SMI) was approved by the China Food and Drug Administration (CFDA) in 1995, which has been widely used to prevent and treat coronary heart disease and chronic HF. SMI is composed of two herbs, including *Panax ginseng* C.A.Mey. and *Ophiopogon japonicus* (Thunb.) Ker Gawl. Wang et al. (2018b) applied iTRAQ-based proteomic approach to identify differentially expressed proteins of SMI, and found that their function was associated with mitochondrial oxidative phosphorylation. SMI significantly increased ATP5D, NDUFB10, and TNNC1 protein expression in rats with myocardial ischemic injury. In vitro, SMI increased ATP and MMP content, and had positive effects on mitochondrial respiration induced by hypoxia. Another trial identified the metabolic effects of SMI against ischemia-reperfusion injury. SMI reduced the mitochondrial mass, enhanced MMP, and inhibited mPTP opening. SMI treatment increased MFN1, MFN2, and OPA mRNA expression, and reduced Drp and Fis mRNA expression. These results mean that the cardioprotective effect of SMI may be associated with mitochondrial dynamics (Yu et al., 2019).

##### Xuesaitong Injection

Xuesaitong injection (XST) is mainly composed of Panax Notoginseng saponins, which has been widely used to prevent and treat cardio-cerebral vascular diseases. XST treatment enhanced the PDH activity, a key enzyme converted pyruvate to acetyl CoA in mitochondria and related to TCA cycle, as well as increased Na^+^-K^+^-ATPase and Ca^2+^-Mg^2+^-ATPase, and elevated intracellular ATP and acetyl-CoA levels in hypoxia/reoxygenation condition. XST significantly enhanced pyruvate dehydrogenase E1 alpha (PDHA1) and ATP synthase 5A (ATP5A) protein expression in H9c2 cells with hypoxia/reoxygenation injury. These proteins are mainly associated with cardiac energy metabolism (Zhao et al., 2017).

##### YiQiFuMai Powder Injection

YiQiFuMai powder injection (YQFM) is designed based on the well-known TCMs prescription Shengmaisan, which is widely applied for the treatment of angina pectoris, coronary heart disease, and chronic heart failure. YQFM is composed of three herbs, including *Panax ginseng* C.A.Mey., *Ophiopogon japonicus* (Thunb.) Ker Gawl, and *Schisandra chinensis* (Turcz.) Baill. YQFM significantly attenuated coronary artery ligation-induced heart failure *via* improving cardiac function and attenuating mitochondrial dysfunction in mice. In addition, YQFM significantly inhibited the Drp1 phosphorylation at Ser616 and increased Mfn2 expression in HF mice and OGD-induced NRVMs injury (Zhang et al., 2019). It indicates that YQFM may improve energy metabolism through regulating mitochondrial dynamics. A different study described in ischemia/reperfusion-Induced myocardial injury, YQFM might regulate energy metabolism through the activation of AMPK phosphorylation (Li et al., 2016a).

## Conclusions and Perspectives

Over the last decade, increasing attention has been focused on the modulation of cardiac energy metabolism as a therapy for the treatment of cardiovascular diseases (Neubauer, 2007). The modulation of cardiac energy metabolism, a complex process involving substrate utilization, mitochondrial oxidative phosphorylation, and ATP transfer and utilization, plays a key pathophysiological role in both cardiac disease progression and its treatment (**Figure 1**). The cardiac metabolic network has complexity and high flexibility in energy substrate utilization during hypoxic/ischemic conditions. In the early stages of cardiac remodeling, alterations in myocardial substrate selection are partly considered to be a compensated and protective mechanism that may impede cardiac irreversible damage. In contrast, in advanced stages, persistent ischemia/hypoxia and subsequent reperfusion may lead to a decrease in fatty acid oxidation and an increase in that of glucose oxidation, which further contributes to lipotoxicity, lactic acidosis, low ATP production, contractile dysfunction, and progression to HF. This process indicates that the relationship between cardiac energy metabolism and IHD is double-sided. Balancing the contradictory effects of energy metabolism at different time points may enhance drug efficacy in the treatment of IHD.

**Figure 3:**
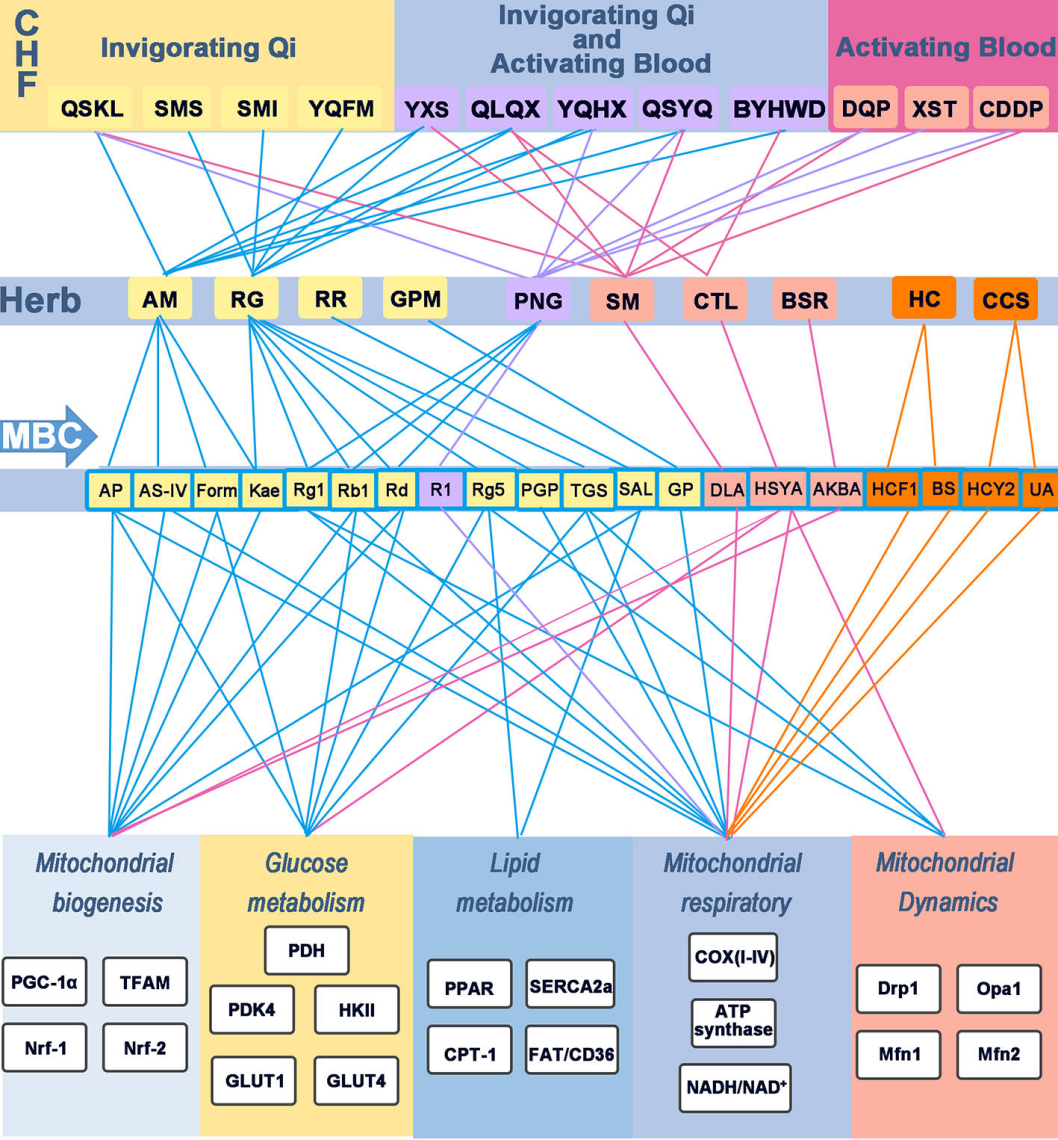
Modulatory effects of CHF, herb, and MBC on energy metabolism in IHD. Chinese herbal formulas, herbs, and the major bioactive component regulate energy metabolism in IHD. Different colored rounded rectangle and lines indicates the different function. The yellow rounder rectangle represents invigorating Qi effects; The purple rounder rectangle represents invigorating Qi and activating Blood effects; The pinkish-orange indicates the formula or herbs with activating Blood; The orange rounder rectangle indicates invigorating Yang effects. Blue lines indicate the relation of herbs with invigorating Qi effects; Purple lines represent the effects of replenishing Qi and activating Blood; Rose lines indicate the effects of activating Blood; And the orange lines represent replenishing Yang. CHF, Chinese herbal formulas; MBC, major bioactive component; COX(I-V), mitochondrial complex (I-V); QSKL, Qishen granules; SMS, Shengmaisan; SMI, Shengmai injection; BYHWD, Buyanghuanwu Decoction; XST, Xuesaitong injection; YXS, Yangxinshi tablet; YQHX, Yiqihuoxue Decoction; YQFM, Yiqifumai powder injection; CDDP, compound Danshen dripping pills; DQP, Danqi pills; AM, *Astragalus membranaceus*; AS-IV, Astragaloside IV; AP, Astragalus polysaccharide; DLA, danshensu; Kae, Kaempferol; Form, Formononetin; RG, *Radix Ginseng*; Rb1, ginsenoside Rb1; Rg1, ginsenoside Rg1; Rg5, ginsenoside Rg5; PNG, *Panax Notoginseng*; R1, Notoginsenoside R1; GPM, *Gynostemma pentaphyllum* (Thunb.) Makino; RR, *Rhodiola rosea L*.; SM, *Salvia miltiorrhiza*; CTL, Carthamus tinctorius L; BSR, Boswellia serrata Roxb.; HC, Herba Cistanches; CCS, *Cynomorium coccineum* subsp.; NADPH oxidase, nicotinamide adenine dinucleotide phosphate-oxidase.

Chinese herbal medicines have great therapeutic potential for the treatment of IHD through the modulation of cardiac metabolism. In this review, we mainly summarize the metabolic effects and the underlying mechanisms of herbs, major bioactive components, and Chinese herbal formulas in IHD. Multiple signal pathways and multiple targets are associated with CHMs-mediated effects on energy metabolism in IHD (**Figure 3**). The details are as follows: (1) The mechanisms of herbs, MBC and CHF in attenuating MI-induced energy metabolism disorder may mainly involve in promoting mitochondrial biogenesis, regulating fatty acid and glucose metabolism, modulating mitochondrial respiratory, and maintaining the balance of mitochondrial dynamics. (2) Chinese herbal formulas that can modulate energy metabolism in IHD usually contain Qi-replenishing and/or Blood-activating herbs. Furthermore, Qi-replenishing and/or Blood-activating CHMs, especially Qi-replenishing herbs and their major components, often play a key role in regulating energy metabolism in IHD. It suggests that the Qi-replenishing effects of CHMs may be related to regulating energy metabolism especially mitochondrial function. (3) Besides, Qi-replenishing herbs or combination of Qi-replenishing and Blood-activating herbs may produce better efficacy on cardiac energy metabolism than monotherapy with Blood-activating herbs. Taking QSYQ as an example, Cui et al. (2018) compared the contribution of five major components (AS-IV, Rb1, Rg1, R1, and DLA) in QSYQ and QSYQ to their potential to regulate energy metabolism in ischemia-induced rat myocardial injury. They found that QSYQ and its five components could improve cardiac structure. Especially, QSYQ significantly improved cardiac function and modulated energy metabolism. The mechanism of QSYQ may prevent ischemia-induced rat myocardial injury by increasing the ATP content, enhancing cTnI and ATP5D expression, and improving the ATP synthase activity. However, five components of QSYQ had different effects on regulating energy metabolism. AS-IV and Rb1 treatment could increase the ATP levels, ATP5D protein expression, and ATP synthase activity, respectively. In contract, R1 only significantly enhanced the cTnI protein expression. Rg1, R1, and DLA of QSYQ had no effects on ATP production, ATP5D expression or ATP synthase activity. These data indicate that the five components in QSYQ exert synergistic effects, promoting ATP production, cTnI and ATP5D expression, and ATP synthase activity. Among them, AS-IV and Rb1 belong to the compounds of Qi-replenishing herbs, whereas R1 and DLA belong to the compounds of Blood-activating herbs. Therefore, the cardioprotective effect of CHMs on energy metabolism in myocardial ischemia may mainly depend on the synergistic effect of combinations of Qi-replenishing and Blood-activating CHMs. Furthermore, studies may provide the structural basis for the effect of major bioactive compounds from CHMs. Major bioactive compounds with energy metabolism regulatory activity identified in CHMs, such as AS-IV, Rb1, Rg1, Rd and R1, mainly belong to the group of saponin compounds that are primarily extracted from *Astragalus membranaceus*, *Panax ginseng* and *Panax notoginseng*, respectively. These possess the ability to regulate mitochondrial biogenesis and mitochondrial respiration through multiple targets and pathways.

Currently, the effects and mechanisms of CHMs on cardiac energy metabolism are still unclear and partially contradictory in experimental studies, which can be primarily attributed to the complex chemical and pharmacological properties of Chinese herbal medicines. The further establishment of the platform of studies on TCM complex prescriptions and their decomposed recipes is essential to clarify the compatibility interactions of different traditional Chinese medicines. Meanwhile, we should pay more attention to the preclinical toxicology study to ensure the herbs’ safety and efficacy of improving energy metabolism in IHD. Cardiac energy metabolism after myocardial ischemia is a dynamic and highly flexible process. The further study needs to compare the change of energy metabolism in different time periods after myocardial ischemia. Additionally, there is a series of complex pathological processes induced by various pathogenetic factors in IHD, which is accompanied by other symptoms and combined with other cardiovascular diseases. However, common animal models are often designed only for a single-factor intervention. Thus, we need to design more combination models such as a rat model of MI combined with diabetes, which are closer to the clinical practice. In clinical trials, although TCMs have a long history of clinical applications in the treatment of IHD, high-quality evidence for their effectiveness is still generally lacking. The clinical research of modern Traditional Chinese Medicine remains many practical problems, including the insufficient understanding of clinical trial registration, the underdevelopment of clinical methodology, imperfect quality control systems, and non-standard clinical research reports (Zhang et al., 2013). Therefore, further clinical studies, including more rigorously designed randomized, double-blind, and large-scale controlled trials are required, and should involve investigating different dosages, administration times, dosing regimens, and delivery routes. Meanwhile, there is a need to urge researchers to perform the international registration of clinical trials, which further improves the experiment design and avoids the selection and reporting bias. The further strengthening assessment of adverse reactions of TCMs is essential to enhance the accuracy of clinical evaluation and reduce the occurrence of adverse drug reactions. Additionally, we must pay more attention to the negative outcomes associated with medication to ensure the integrity and facticity of TCMs results in clinical research. Currently, most clinical studies of TCM prescriptions in IHD often use the placebo as a control group and lack comparisons to clinical drugs. Thus, the design of clinical research needs to compare the western medicine control group to evaluate the efficacy of TCM. The characteristics of TCM such as the theory of treatment based on syndrome differentiation also increase the complexity and difficulty of clinical research. It is necessary to further establish a new qualitative and quantitative research of the mixed-methods approach to adapt for the characteristics of traditional Chinese Medicine.

## Author Contributions

FL conceived the topic and wrote the manuscript. JL and SL helped to revise the manuscript and draw the figure and consulted the references. SG and PL revised and modified the manuscript.

## Funding

This work is supported by the National Natural Science Foundation of China (No. 81473552), and the Postdoctoral Science Foundation of China (No. 2019TQ0043).

## Conflict of Interest

The authors declare that the research was conducted in the absence of any commercial or financial relationships that could be construed as a potential conflict of interest.
